# Use of imaging and clinical data to screen for cardiovascular disease in asymptomatic diabetics

**DOI:** 10.1186/s12933-016-0334-4

**Published:** 2016-02-09

**Authors:** Carlos Henrique Reis Esselin Rassi, Timothy W. Churchill, Carlos A. Fernandes Tavares, Mateus Guimaraes Fahel, Fabricia P. O. Rassi, Augusto H. Uchida, Bernardo L. Wajchenberg, Antonio C. Lerario, Edward Hulten, Khurram Nasir, Márcio S. Bittencourt, Carlos Eduardo Rochitte, Ron Blankstein

**Affiliations:** Heart Institute (InCor), University of São Paulo, Medical School, Brazil, Av. Dr. Enéas de Carvalho Aguiar, 44, Andar AB, Cerqueira César, São Paulo, SP, 05403-000 Brazil; Department of Medicine, Brigham and Women’s Hospital, 75 Francis St, Boston, MA 02115 USA; Division of Cardiovascular Medicine, Brigham and Women’s Hospital, 75 Francis St, Boston, MA 02115 USA; Department of Cardiology, Baptist Health South Florida, 8900 N. Kendall Drive, Miami, FL 33176 USA; Center for Clinical and Epidemiological Research, Division of Internal Medicine, University Hospital, and State of São Paulo Cancer Institute (ICESP), University of São Paulo, São Paulo, Brazil

**Keywords:** Type 2 diabetes mellitus, Coronary artery disease, Coronary computed tomography angiography

## Abstract

**Background:**

There is increasing evidence to suggest that not all individuals with type 2 diabetes mellitus (T2DM) have equal risk for developing cardiovascular disease. We sought to compare the yield of testing for pre-clinical atherosclerosis with various approaches.

**Methods:**

98 asymptomatic individuals with T2DM without known coronary artery disease (CAD) were enrolled in a prospective study and underwent carotid ultrasound, exercise treadmill testing (ETT), coronary artery calcium (CAC) scoring, and coronary computed tomography angiography (CTA).

**Results:**

Of 98 subjects (average age 55 ± 6, 64 % female), 43 (44 %) had coronary plaque detectable on CTA, and 38 (39 %) had CAC score >0. By CTA, 16 (16 %) had coronary stenosis ≥50 %, including three subjects with CAC = 0. Subjects with coronary plaque had greater prevalence of carotid plaque (58 % vs. 38 %, p = 0.01) and greater carotid intima media thickness (0.80 ± 0.20 mm vs. 0.70 ± 0.11 mm, p = 0.02). Notably, 18 of 55 subjects (33 %) with normal CTA had carotid plaque. Eight subjects had a positive ETT, of whom five had ≥ 50 % coronary stenosis, two had <50 % stenosis, and one had no CAD. Among these tests, CAC scoring had the highest sensitivity and specificity for prediction of CAD.

**Conclusion:**

Among asymptomatic subjects with T2DM, a majority (56 %) had no CAD by CTA. When compared to CTA, CAC was the most accurate screening modality for detection of CAD, while ETT and carotid ultrasound were less sensitive and specific. However, 33 % of subjects with normal coronary CTA had carotid plaque, suggesting that screening for carotid plaque might better characterize stroke risk in such patients.

## Background

Cardiovascular disease is the leading cause of morbidity and mortality among individuals with type 2 diabetes mellitus (T2DM) [1–3], and diabetics have a higher risk of cardiovascular disease as compared to non-diabetics with a similar risk factor burden [4]. Moreover, once individuals with diabetes present with a coronary heart disease event, they experience a worse prognosis than non-diabetics [5–8]. Unfortunately, diabetics are also known to have a high burden of asymptomatic cardiovascular disease. Thus, a wide variety of clinical strategies have been proposed for identifying diabetics with coronary artery disease (CAD) before it becomes clinically manifest.

Although screening programs are currently employed in asymptomatic diabetics in some clinical settings, their potential capability to reduce the rate of adverse cardiac outcomes remains unproven [9, 10]. In part, this may be due to lower than anticipated event rates in clinical trials, as well as widespread use of preventive therapies irrespective of the screening strategy used [11]. While the available studies to date support the use of preventive therapies for most patients with diabetes, the yield and optimal technique for risk stratifying lower-risk individuals with diabetes is uncertain. Despite the lack of proven benefit, there may remain a role for screening selected individuals with diabetes, particularly if the test results could favorably influence downstream medical and lifestyle therapies. In addition, such screening approaches could be useful to provide more individualized assessment when deciding on the intensity of statin therapy, the role of aspirin [12], or potentially in the future, the role of anti-inflammatory therapies [13] and newer lipid lowering agents [14].

Multiple imaging and laboratory techniques are available to detect the presence of pre-clinical disease and consequently characterize an individual’s risk of future cardiovascular events. However, it remains uncertain how each of these approaches compare to each other and to more expensive imaging techniques in the risk stratification of individuals with T2DM. Therefore, using coronary computed tomography angiography (CTA) as the reference standard, we sought to compare the use of coronary artery calcium (CAC) scoring, carotid ultrasound, and exercise treadmill testing (ETT) for detecting sub-clinical CAD among a cohort of asymptomatic patients with diabetes.

## Methods

### Patient enrollment

In this prospective cohort study, 98 asymptomatic subjects with T2DM, as defined by American Diabetes Association criteria [15], were recruited between June 2011 and January 2013 from the Endocrinology Outpatient Clinic of the University of São Paulo School of Medicine Clinics Hospital. The Ethics Committee of the University of São Paulo approved the study, and all patients provided informed consent during their initial visit.

We included individuals aged 40–65 years with a known duration of diabetes of less than 10 years and without known prior cardiovascular disease. Exclusion criteria included a history of heart failure, ischemic heart disease, chest pain, angina, arrhythmia, severe hypertension (blood pressure >180/100 mmHg), renal or hepatic failure, dyspnea at rest, total cholesterol >350 mg/dL, low-density lipoprotein (LDL) cholesterol >250 mg/dL, triglycerides >500 mg/dL, body mass index (BMI) >45 kg/m2, known neoplasm, pregnancy, dementia, and an allergy to iodinated contrast.

Power calculation [16, 17] estimates that a sample size of 51 patients would be required for an α of 0.05 and power of 0.8 in order to detect a difference among the sensitivity of the three screening tests, assuming CAC sensitivity to be 90 % [18, 19], ETT sensitivity 30 % [20], and carotid ultrasound sensitivity 50 % [21, 22] for detection of any coronary plaque. ETT has highly variable previously reported sensitivities for detection of obstructive CAD, but to screen for the presence of any plaque, including non-obstructive, the sensitivity of ETT is lower [20].

### Clinical and laboratory evaluation

All clinical and historical data (e.g. diabetes duration) were prospectively collected by a study physician prior to all imaging and laboratory test results. Laboratory tests included fasting glucose and glycosylated hemoglobin, total cholesterol and cholesterol fractions, triglycerides, creatinine, liver function tests, blood counts, and urine and serum human chorionic gonadotropin β (in women of childbearing age). Microalbuminuria was measured using 24-hour urine collection.

### Coronary CTA acquisition and analysis

Coronary CTA and CAC calcium scanning were performed using Toshiba Aquillion One scanner with 320 detectors, 0.5 mm slice thickness, with gantry rotation of 350 ms. Scan coverage in the z-axis ranged from 12 to 16 cm. For the CTA acquisition, we used tube voltage between 80–135 kV and tube current between 200–580 mA, both selected according to patient BMI. Prior to each scan, the patient’s blood pressure and heart rate were assessed, and if the heart rate was above 70 beats per minute, beta-blockers were administered orally. Following oral beta blocker administration, if the heart rate was greater than 64 bpm, intravenous metoprolol was administered. We administered 70–100 mL of iodinated contrast (Iopamiron 370 mg/ml; Bayer Schering Pharma, Berlin, Germany) via an automated injector at a rate of 5 mL/second. The estimated mean radiation dose for the complete CT protocol (coronary CTA plus CAC scanning) was 7.1 mSv per patient.

All coronary CTA images were transferred to a workstation (Vitrea FX—Vital Image) and analyzed by two experienced cardiac imagers who were blinded to all other data. A standard 18-segment coronary tree model was used [23]. The calcium was calculated according to the Agatston protocol [24].

CAD was defined by the presence of any atherosclerotic plaque, which was defined as a tissue structure >1 mm^2^ that was contained within and/or adjacent to the coronary artery lumen and could be clearly distinguished from the vessel lumen [25]. Plaques were classified according to the degree of luminal obstruction. Obstructive CAD was defined as the presence of at least one plaque causing a luminal reduction of more than 50 % [23]. In cases of a disagreement between the two examiners, a third experienced cardiologist mediated a consensus.

### Other testing modalities

In addition to CAC scoring and CTA analysis, all subjects underwent exercise treadmill testing and carotid ultrasonography. Exercise treadmill tests were interpreted by an experienced cardiologist who was blinded to all other test results. When considering the exercise protocol, 42 % of subjects exercised using the Bruce protocol, and 52 % with the modified Bruce protocol. Of the remaining six subjects, five underwent test with Ellestad protocol and one with ramp protocol. The ECG was defined as positive if there were horizontal or downsloping ST depressions greater than 1 mm in two contiguous leads (except aVR) 80 ms after the J-point. The ETT was considered non-diagnostic if subjects did not achieve 85 % of the age predicted maximum heart rate.

Carotid ultrasounds were interpreted by an experienced radiologist. Carotid intima media thickness (CIMT) was manually measured in both carotid arteries at end diastole over a 1 cm segment of the common carotid artery located 0.5 cm below the carotid-artery bulb [26]. CIMT ≥ 1 mm was defined as abnormal [27]. Carotid plaque was defined as a focal region protruding into the vessel lumen that had either CIMT ≥ 1.5 mm or focal wall thickening at least 50 % greater than that of the surrounding vessel wall [28].

### Statistical analysis

Continuous variables are presented as mean and standard deviation or as median and quartiles, as appropriate, and tested for significance using two-tailed *t* test or Kruskal–Wallis test depending on whether the distribution was normal. Categorical variables are presented as absolute values and proportions and tested for significance with a Chi squared test. To compare the diagnostic yield of various screening approached to detect CAD, we calculated sensitivity and specificity for each test, using the presence of any disease by CTA as the reference standard. All statistical analysis was done using Stata 12.1 (StataCorp, College Station, Texas).

## Results

### Patient characteristics

Characteristics of study subjects are shown in Table 1, stratified by the presence of any CAD. Mean age was 54.5 years, and 63 (63 %) of subjects were female. The mean hemoglobin A1c was 7.3 % and 22 (22 %) were on insulin therapy. The mean LDL cholesterol was 116.7 mg/dL. Fewer than 50 % of subjects were on statin therapy or aspirin, and 50 (51 %) were on an ACE inhibitor or an ARB. The median 10-year risk was 13.0 % as assessed by Framingham Risk Score for coronary heart disease, 8.2 % by the 2013 American Heart Association risk calculator, and 11.4 % by United Kingdom Prospective Diabetes Study (UKPDS) risk engine.

**Table 1:** Patient characteristics

	All subjects n = 98	Coronary artery disease n = 43 (43.9 %)	No coronary artery disease n = 55 (56.1 %)	p Value
Demographics				
Age, years	54.5 ± 6.1	56.5 ± 5.8	53.0 ± 5.9	<0.01
Female sex (#, %)	63 (64.3 %)	24 (55.8 %)	39 (70.9 %)	0.12
Race (#, %)				
Caucasian	64 (65.3 %)	33 (76.7 %)	31 (56.4 %)	0.03
Black	19 (19.4 %)	4 (9.3 %)	15 (27.3 %)	
Asian	7 (7.1 %)	1 (2.3 %)	6 (10.9 %)	
Other	8 (8.2 %)	5 (11.6 %)	3 (5.5 %)	
Clinical data				
Body mass index	29.4 ± 4.7	29.5 ± 4.8	29.3 ± 4.7	0.83
Abdominal circumference, cm	103.0 ± 12.1	105.0 ± 12.0	101.4 ± 12.1	0.15
Systolic blood pressure, mmHg	120.9 ± 17.2	125.3 ± 17.3	117.4 ± 16.5	0.02
Diastolic blood pressure, mmHg	71.9 ± 11.9	73.9 ± 12.3	70.3 ± 11.4	0.15
Treatment for hypertension (#, %)	64 (65.3)	34 (79.1 %)	30 (54.5 %)	0.01
Duration of diabetes, years	5.2 ± 3.3	6.5 ± 3.2	4.2 ± 3.0	<0.01
History of hyperlipidemia (#, %)	55 (56.1 %)	26 (60.5 %)	29 (52.7 %)	0.44
Family history of coronary artery disease (#, %)	10 (10.2 %)	7 (16.3 %)	3 (5.5 %)	0.08
Smoking status (#, %)				
Current smoker	20 (20.4 %)	10 (23.3 %)	10 (18.2 %)	0.74
Former smoker	8 (8.2 %)	4 (9.3 %)	4 (7.3 %)	
Never smoker	70 (71.4 %)	29 (67.4 %)	41 (74.5 %)	
Framingham risk score (10-year estimated risk) (median, interquartile range)	13.0 % (8.0–16 %)	13.0 % (10–20 %)	13.0 % (8–16 %)	0.07
2013 AHA/ACC risk calculator (10-year risk of atherosclerotic cardiovascular disease) (median, interquartile range)	8.2 % (4.0–16.0 %)	13.5 % (5.5–19.7 %)	7.0 % (2.3–13 %)	<0.01
UKPDS risk engine 10-year predicted risk of coronary heart disease (median, interquartile range)	11.4 % (5.2–19.4 %)	17.9 % (11.7–26.2 %)	7.2 % (3.8–11.6 %)	<0.01
Laboratory data				
Hemoglobin A1c	7.3 ± 1.7 %	8.0 ± 1.7 %	6.8 ± 1.5 %	<0.01
Total cholesterol, mg/dL	193.5 ± 39.7	198.5 ± 46.6	189.7 ± 33.3	0.28
HDL cholesterol, mg/dL	45.5 ± 13.2	46.7 ± 14.3	44.5 ± 12.5	0.44
LDL cholesterol, mg/dL	116.7 ± 35.1	120.6 ± 41.4	113.7 ± 29.2	0.33
Triglycerides, mg/dL (median, interquartile range)	133 (103–198)	148 (101–213)	129 (103–194)	0.46
Microalbuminuria, mg/24 h (median, interquartile range)	5.8 (3.9–11.8)	8.1 (3.9–18.7)	5.4 (3.7–9.0)	0.09
Medications				
Insulin use (#, %)	22 (22.5 %)	15 (34.8 %)	7 (12.7 %)	<0.01
Oral hypoglycemic (#, %)	82 (83.7 %)	39 (90.7 %)	43 (78.2 %)	0.10
ACE or ARB (#, %)	50 (51.0 %)	27 (62.8 %)	23 (41.8 %)	0.04
Statin (#, %)	45 (45.9 %)	25 (58.1 %)	20 (26.4 %)	0.03
Aspirin (#, %)	33 (33.7 %)	19 (44.2 %)	14 (25.5 %)	0.052

Values given are mean ± standard deviation unless otherwise specified

p Values were calculated using two-tailed t test and Chi squared test; Kruskal–Wallis test was used for comparison of medians

### Characteristics of patients with coronary artery disease

Subjects with coronary disease on coronary CTA were more likely to be older and have hypertension, and reported a longer duration of T2DM than subjects without CAD. Among laboratory parameters, only hemoglobin A1c was significantly higher among those with CAD on CTA. Insulin use was more common among those with CAD. Similarly, use of cardiovascular medications, including ACE inhibitors or ARBs and statins, was more common in subjects with CAD. While there was a trend towards increased aspirin use among those with CAD, this did not reach statistical significance (p = 0.052).

### Coronary CTA findings

Results of the coronary CTA are shown in Table 2. Overall, 55 subjects (56 %) had no CAD, while 27 (28 %) had non-obstructive plaque (<50 % stenosis). The remaining 16 subjects (16 %) had at least one coronary stenosis over 50 %. Among the 43 subjects with any plaque, 27 (63 %) had disease in multiple coronary vessels, and over one third (16 subjects, or 37 %) had disease involving greater than four coronary segments. Multivessel obstructive disease (defined as greater than 50 % stenosis in more than one coronary artery) was uncommon; only three subjects had obstructive disease in two arteries and one in all three arteries.

**Table 2:** Coronary computed tomography angiography findings

Luminal stenosis on coronary CTA	Number of subjects (%)
No stenosis	55 (56.1)
1–24 %	13 (13.3)
25–49 %	14 (14.3)
50–69 %	7 (7.1)
70 % or greater	9 (9.2)
Number of coronary segments with plaque	Number of subjects (%)
0 segments	55 (56.1)
1–4 segments	27 (27.6)
5 or more segments	16 (16.3)
Number of coronary arteries with plaque	Number of subjects (%)
No plaque	55 (56.1)
1 vessel	16 (16.3)
2 vessels	15 (15.3)
3 vessels	12 (12.2)
Number of coronary arteries with obstructive disease (≥50 % stenosis)	Number of subjects (%)
No obstructive disease	82 (83.7)
1 vessel	12 (12.2)
2 vessels	3 (3.1)
3 vessels	1 (1.0)
Coronary artery calcium (Agatston score)	Number of subjects (%)
0	60 (61.2)
1–99	24 (24.5)
100 or greater	14 (14.3)

### CAC findings

Overall, 60 subjects (61 %) did not have any CAC (Table 2). Among the remaining 38 subjects with CAC, the majority (24 subjects, or 63 %) had Agatston score <100, with the remaining 14 (37 %) having an Agatston score of 100 or greater.

Only five subjects had plaque on coronary CTA despite a CAC of zero. The number of affected coronary segments in these subjects ranged from 1 to 5, and three of the five individuals had stenosis greater than 50 %. Two of these five subjects had carotid plaque, and one had a positive ETT.

### Results of carotid ultrasound and exercise treadmill testing

Results from carotid ultrasounds and ETT, stratified by the presence of CAD, are presented in Table 3. Mean CIMT was greater in subjects with CAD. Similarly, a greater proportion of subjects with CAD had carotid plaque than those who did not have any CAD.

**Table 3:** Results of screening tests stratified by the presence or absence of coronary artery disease

Screening tests	All subjects (n = 98)	Subjects with coronary artery disease (n = 43)	Subjects without coronary artery disease (n = 55)	p Value
Coronary artery calcium				
0	60 (61.0 %)	5 (11.6 %)	55 (100 %)	<0.01
≥ 1 (Agatston score)	38 (38.8 %)	38 (88.4 %)	0 (0 %)	
Carotid artery ultrasound				
Maximum intima medial thickness (IMT), mm	0.75 ± 0.16	0.80 ± 0.20	0.70 ± 0.11	<0.01
IMT ≥ 1.0 mm (#, %)	11 (11.2 %)	11 (25.6 %)	0	<0.01
Carotid plaque (#, %)				
No carotid plaque	55 (56.1 %)	18 (41.9 %)	37 (67.3 %)	0.01
Carotid plaque	43 (43.9 %)	25 (58.4 %)	18 (32.7 %)	
Carotid plaque or IMT ≥ 1.0 mm	44 (44.9 %)	26 (60.5 %)	18 (32.7 %)	<0.01
Exercise treadmill test				
ECG test results (#, %)				
Negative	69 (70.4 %)	26 (60.5 %)	43 (78.2 %)	0.04
Positive	8 (8.2 %)	7 (16.3 %)	1 (1.8 %)	
Negative with <85 % MPHR	20 (20.4 %)	10 (23.3 %)	10 (18.2 %)	
Inconclusive due to LBBB	1 (1.0 %)	0	1 (1.8 %)	
Specific test outcomes				
METS	8.5 ± 2.1	8.2 ± 1.8	8.7 ± 2.4	0.19
Duke treadmill score	8.3 ± 4.8	7.1 ± 5.0	9.2 ± 4.4	0.03

p Values calculated using two-tailed t test, Chi squared test, and Kruskal–Wallis test

*MPHR* maximal predicted heart rate

Only 8 (8 %) subjects had positive ECG changes during the exercise treadmill test, although 20 % of subjects did not reach maximal predicted heart rate and one subject had a non-diagnostic test due to baseline left-bundle branch block. Two patients had angina during the exercise treadmill test. Of these, one had ECG changes and considered as a positive test; the other had atypical angina without ECG changes and was considered as a negative test. The number of METS achieved during the test did not differ between the CAD and the non-CAD group (8.2 METs vs. 8.7 METS, p = 0.19), though subjects with CAD had a lower Duke Treadmill Score than those without CAD (7.1 vs. 9.2, p = 0.03).

### Predictive value of screening tests and historical parameters for coronary artery disease

**Fig. 1:**
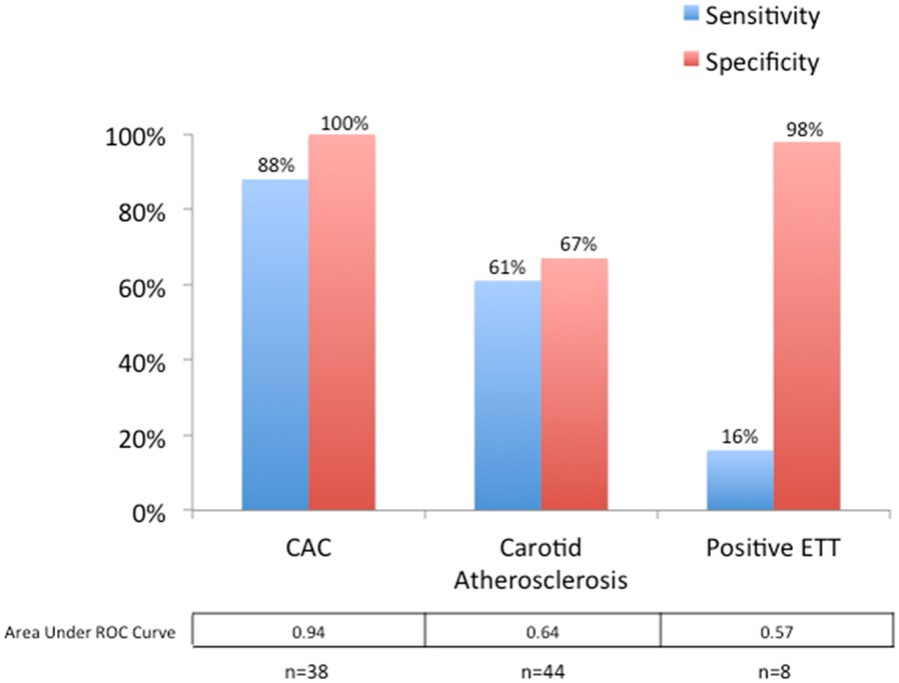
Sensitivity and specificity and area under receiver operating characteristics curves of different screening modalities for detection of coronary artery disease as diagnosed by CTA. CAC scoring was the most sensitive and specific test for detection of CAD, with the greatest area under the ROC curve. Carotid atherosclerosis was defined as presence of carotid plaque or CIMT ≥ 1 mm

The sensitivity and specificity for each testing modality for the prediction of CAD are shown in Fig. 1. CAC offered the best combination of sensitivity and specificity of any evaluated test to detect CAD. Carotid disease, by contrast, was only modestly predictive of the presence of coronary disease, with sensitivity and specificity in the 60 % range. ETT, in turn, was not sensitive, but had very high specificity. Area under receiver operating characteristics (ROC) curves for these three modalities were 0.94 for CAC, 0.64 for carotid ultrasound, and 0.57 for ETT (Fig. 1); p value was <0.01 for comparison of CAC with other modalities, but was non-significant (0.29) for comparison of carotid ultrasound with ETT.

Test characteristics of evaluated historical parameters are shown in Table 4. Of these, hypertension and hyperlipidemia were the most sensitive, while factors relating to severity of diabetes—insulin use, poor glycemic control, and long duration of diabetes—all were relatively insensitive but considerably more specific. Combinations of these parameters yielded decreasing sensitivity and increasing specificity.

**Table 4:** Test characteristics of clinical data for prediction of coronary artery disease detected by coronary CTA

	Definition of positive	Frequency (total n = 98) (%)	Sensitivity (%)	Specificity (%)
Clinical variables				
Age ≥50 years	Age ≥50 years	75 (77)	83.7	29.1
Age ≥55 years	Age ≥55 years	44 (44.9)	60.5	67.3
High risk clinical criteria				
Hypertension	History of hypertension, current treatment, or SBP ≥ 140 mmHg	65 (66.3)	81.4	45.5
Hyperlipidemia	LDL cholesterol ≥130 mg/dL or statin therapy	65 (66.3)	76.7	41.8
Insulin use	Current insulin use at time of CTA	22 (22.5)	34.9	87.3
Poor glycemic control	Hemoglobin A1c ≥ 8.0 %	30 (30.6)	46.5	81.8
Long duration of diabetes	Duration of DM > 6 years	33 (33.7)	51.2	80.0
Combinations of high risk clinical criteria				
At least 1 high risk clinical criterion	≥1 criterion defined above	87 (88.8)	95.3	16.4
At least 2 high risk clinical criteria	≥2 criteria defined above	65 (66.3)	86.0	49.1
At least 3 high risk clinical criteria	≥3 criteria defined above	38 (38.8)	65.1	81.8
At least 4 high risk clinical criteria	≥4 criteria defined above	15 (15.3)	27.9	94.5
At least 5 high risk clinical criteria	5 criteria defined above	8 (8.2)	16.3	98.2

Coronary artery disease defined as the presence of any coronary artery plaque on CTA

**Fig. 2:**
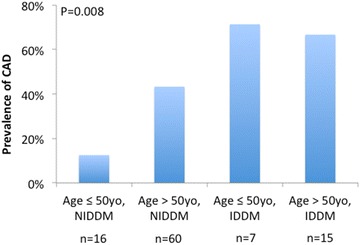
Prevalence of coronary artery disease as detected by coronary CTA stratified by insulin use and age. The prevalence of CAD was greater in those over age 50 and those with insulin-dependent diabetes

**Fig. 3:**
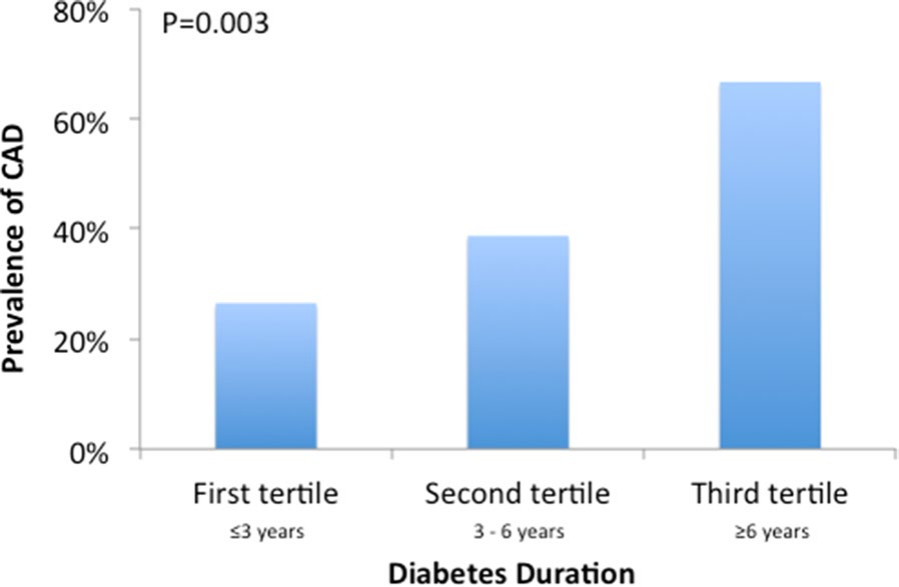
Prevalence of coronary artery disease as detected by coronary CTA stratified by duration of diabetes. The prevalence of CAD increased with increasing duration of diabetes, from 26 % in those with diabetes for 3 year or less to 67 % in those with diabetes for 6 years or longer

The prevalence of CAD, stratified by insulin use and subject age, is presented in Fig. 2. Among non-insulin dependent diabetics, younger subjects had a lower prevalence of CAD. However, among insulin-dependent subjects, there was no significant difference in CAD prevalence by age. The prevalence of CAD also increased by duration of diabetes, as shown in Fig. 3.

## Discussion

In this study of asymptomatic diabetic subjects without known cardiovascular disease, we showed that there is a substantial heterogeneity in the prevalence of subclinical coronary disease, as only 44 % of subjects had evidence of any plaque by coronary CTA and only 16 % had obstructive CAD. When coronary CTA was used as the reference standard, CAC had the highest sensitivity for detection of coronary plaque, while carotid ultrasound, exercise treadmill testing, and risk factors, either alone or in combination, had lower sensitivity and specificity. Finally, we also found that 33 % of the patients who did not have coronary plaque on CTA had atherosclerotic plaque involving the carotid arteries.

### Prevalence of subclinical coronary disease

Perhaps more notable than the 44 % of subjects with coronary disease is the fact that 56 % of the study participants had no atherosclerotic plaque in their coronary arteries. Coronary CTA is a highly sensitive measure for detection of CAD [29, 30], and subjects without plaque on CTA correspondingly have a low rate of cardiovascular events and represent a low risk group [31–33]. This finding demonstrates that there is considerable variability in cardiac risk amongst diabetics and brings into question the classification of diabetes as a coronary heart disease equivalent [34]. As a risk stratification tool, screening coronary CTA represents an efficient way to define a subset of diabetics at much lower risk for coronary heart disease, with a number needed to screen of two in order to detect one low risk patient in our population. Nevertheless, further data are needed regarding the role of preventative therapies (or conversely safety of withholding such therapies) in individuals who have T2DM without any atherosclerosis on CTA [35, 36].

The proportion of subjects with normal coronary arteries was greater in our population than in most published series, which have reported prevalence rates of normal CTA in individuals with diabetes ranging from 13–34 % of subjects [31, 37–40]. Most published data, however, pertain to subjects referred for coronary CTA, who had either symptoms concerning for obstructive CAD or an abnormal prior stress test. However, even when we compared the prevalence of normal CTA in our study to other screening populations of asymptomatic diabetics, we observed a higher rate in our population. For example, in the FACTOR 64 trial, a large, multi-center study of coronary CTA in asymptomatic diabetics, 31 % of subjects had a normal CTA. This difference likely reflects the fact that FACTOR 64 enrolled a population of diabetics with higher cardiovascular risk—when compared to our study, the FACTOR 64 population had a greater proportion of men, an older cohort (62 vs. 55 years in our study), a longer duration of diabetes (13.9 vs. 5.2 years), and a higher rate of insulin use (43 vs. 22 %) [41].

### Screening modalities for coronary disease

Among the screening modalities tested in our study, we found that CAC scoring had the greatest sensitivity and specificity for detection of CAD. The strong test characteristics of calcium scoring are unsurprising, as CAC is by definition 100 % specific for coronary plaque and the sensitivity is only limited by the presence of exclusively non-calcified plaque.

In our sample, the subset whose CAD would be missed by calcium scoring—those with coronary plaque but without coronary calcium—represented 5 % of the total population and 8 % of those with a calcium score of zero. Other authors have found higher rates of coronary plaque in such patients without any coronary calcium [42]. However, since most prior studies have demonstrated an extremely low event rate for patients with CAC scores of zero, it is unclear whether the presence of exclusively non-calcified plaque is associated with any meaningful increase in cardiovascular risk [43].

By contrast, exercise treadmill testing was insensitive, but quite specific for the presence of coronary disease, a conclusion that is in keeping with prior studies of ETT in diabetic populations [44–47]. This result is consistent with our pathophysiologic understanding of exercise testing, as a positive ETT requires the presence of flow-limiting coronary disease, which implies a more advanced coronary lesion. The proportion of subjects with non-diagnostic testing is also in-line with prior series [44, 48].

Carotid ultrasound had only modest results in both sensitivity and specificity, suggesting that this is a less useful test for the evaluation of possible coronary heart disease in this population. Similar to our findings, Djaberi et al. [49] evaluated 150 asymptomatic diabetics, using a lower CIMT cutoff of 0.62 mm, and reported a sensitivity and specificity of 76 and 71 %, respectively for prediction of any coronary atherosclerosis . Other data, mostly in non-diabetic cohorts, have varied on this subject. Guarici and colleagues [50], in a moderate risk group of symptomatic patients, were able to demonstrate CIMT to be independently predictive of obstructive coronary plaque. Other series, by contrast, have not found any association between carotid IMT and the presence of coronary atherosclerosis by CTA [51]. While there are data supporting the association between carotid disease (both increased CIMT and carotid plaque) and coronary heart disease events [52–55], the presence of carotid disease likely has a stronger association with stroke; data from MESA support this, demonstrating that CAC was a much stronger predictor of coronary events than carotid ultrasound measures [56].

### Carotid disease in the absence of CAD

Interestingly, almost one-third of subjects in our study who did not have CAD were found to have carotid artery plaque on carotid ultrasound. This represents a potentially intriguing subgroup, as these subjects are low risk—by virtue of their absence of CAD—from a coronary perspective but may have elevated cerebrovascular risk [57], especially since diabetes itself is also a risk factor for cerebrovascular disease [58]. A similar population of patients—with carotid atherosclerosis but without coronary disease—was described in a recent paper by Cohen et al. [59] who demonstrated that, in a cohort of 150 predominantly non-diabetic patients referred for coronary CTA, 52 % (33 of 63) of those without any CAD on coronary CTA had carotid plaque.

The exact clinical implications of this finding, however, remain unclear, and data are mixed as to whether carotid plaque represents a significant cerebrovascular risk factor in diabetics above and beyond traditional cardiovascular risk factors, as associations between carotid plaque and stroke often do not retain statistical significance in multivariate models or do so only in certain subsets of patients [27, 60, 61]. Carotid atherosclerosis may also herald atherosclerosis in other vascular beds, and this population may be at increased risk of developing incident cardiovascular disease other than stroke. Thus, future studies are warranted regarding the prognostic implications of having carotid plaque in the absence of coronary atherosclerosis.

### Other markers of risk

Our data also show higher prevalence of CAD in subjects on insulin therapy and an association between the presence of CAD and both insulin use and duration of diabetes. Subjects receiving insulin and those who had carried a diagnosis of diabetes for a longer period of time had greater prevalence of CAD, and insulin use was the most specific single clinical characteristic for the presence of coronary disease. Insulin use has previously been associated with a greater extent of coronary disease in diabetics [31, 62], and duration of diabetes has also been associated with worse clinical outcomes [63]. However, these two characteristics are rarely highlighted as part of a practical, clinical approach to risk stratification, despite the presence of a potential pathophysiological link and potential clinical utility of those findings [62].

### Screening in asymptomatic diabetics

Despite the inherent appeal of early detection of CAD, studies of screening for coronary disease in asymptomatic diabetics have thus far not shown a convincing benefit. The Detection of Ischemia in Asymptomatic Diabetics (DIAD) study, the first large-scale, randomized trial to assess a screening program in asymptomatic diabetics, did not find a difference in adverse cardiac outcomes between screened and unscreened subjects [9]. Importantly, though, this study screened subjects with SPECT myocardial perfusion imaging, a test less sensitive than coronary CTA for detection of CAD, and rates of modern medical therapy (statins, aspirin, beta blockers, ACE inhibitors) were high and similar in both groups.

The more recent FACTOR-64 trial addressed this limitation, using coronary CTA to screen asymptomatic diabetics, but similarly did not demonstrate a beneficial effect of screening on hard cardiovascular outcomes [41]. In this case, just as with DIAD, one of the most salient features of the trial was again the low event rate—less than 2 % annually—which was substantially less than the 16 % event rate over 2 years that had been anticipated for the study’s power calculation. This difference reflected the excellent background medical therapy, with ~75 % of study participants having an LDL cholesterol <100 mg/dL at baseline.

According to the 2013 American College of Cardiology/American Heart Association cholesterol guidelines, all diabetics—and therefore all subjects in our study—should be treated with a moderate or high-intensity statin [64]. However, statin therapy is not without adverse effects [65–68], and it is conceivable that subjects without coronary disease may not experience sufficient benefit of statin therapy, although further research is required in this area. Specifically, what the DIAD and FACTOR-64 studies did not address was whether subjects with negative screening tests might safely avoid some preventative therapies. Within our cohort, screening with coronary CTA identified over 50 % of subjects as having low risk for coronary heart disease, suggesting that a significant proportion of individuals who might choose, based on their preferences, to focus on lifestyle therapies while deferring pharmacological treatments. Nevertheless, further studies are needed regarding the role of statin therapy to reduce the lifetime risk of future cardiovascular events in individuals who do not have evidence of coronary plaque.

### Limitations

Our study is limited by its small size, composed of 98 individuals. However, we examined a homogenous and well-defined cohort, consisting only of asymptomatic subjects with diabetes who had no known cardiovascular or renal disease. In addition, despite its small size, our study is unique as it is the only study of which we are aware that has correlated the findings of CAC scoring, carotid ultrasonography, ETT and biochemical testing against the gold standard of coronary CTA in asymptomatic individuals with diabetes. We did not incorporate data on visceral adiposity or the relative severity of body fat, both of which have been shown to be associated with subclinical atherosclerosis [69, 70]. Finally, another limitation is that our data are cross-sectional and lack information on clinical outcomes. However, it is well documented that CAC scoring and the presence of CAD by CTA are strong predictors of cardiovascular events among subjects with diabetes [31, 71–76].

## Conclusion

Within this population of asymptomatic subjects with diabetes, 56 % did not have any atherosclerotic coronary artery disease by coronary CTA, a finding that highlights the heterogeneity of cardiovascular risk in diabetics. Further research is required to better delineate whether there is any clinical role for screening programs to detect such individuals. Such screening could be used to individualize the intensity of lifestyle and pharmacotherapy based on risk level as well as patient preferences. When compared to coronary CTA, screening for CAC was the most accurate method for detection of subclinical coronary artery disease, as compared to carotid ultrasonography or exercise treadmill testing. Finally, a substantial proportion of subjects without CAD had detectable carotid artery plaque, and further investigation is needed to understand both if this represents a population at increased risk of adverse events and whether any interventions might decrease this risk.

## Abbreviations

BMI: body mass index
CAC: coronary artery calcium
CAD: coronary artery disease
CIMT: carotid intima media thickness
CTA: computed tomography angiography
ECG: electrocardiogram
ETT: exercise treadmill testing
T2DM: type 2 diabetes mellitus
